# Laparoscopic anatomical partial splenectomy for extremely rare isolated splenic lymphangioma in an adult: a case report and literature review

**DOI:** 10.3389/fonc.2024.1460566

**Published:** 2024-09-04

**Authors:** Ruizi Shi, Pei Yang, Yangjie Guo, Yiping Tang, Hua Luo, Chuan Qin, Ting Jiang, Yu Huang, Ziqing Gao, Xintao Zeng, Jianjun Wang

**Affiliations:** ^1^ Department of Hepatobiliary Surgery, Mianyang Central Hospital, School of Medicine, University of Electronic Science and Technology of China, Mianyang, China; ^2^ Department of Pathology, Mianyang Central Hospital, School of Medicine, University of Electronic Science and Technology of China, Mianyang, China; ^3^ NHC Key Laboratory of Nuclear Technology Medical Transformation, Mianyang Central Hospital, School of Medicine, University of Electronic Science and Technology of China, Mianyang, China

**Keywords:** splenic benign tumor, splenic lymphangioma, laparoscopic surgery, case report, anatomical resection

## Abstract

**Background:**

Benign tumors of the spleen are rare compared to those of other parenchymal organs, accounting for less than 0.007% of all tumors, and are often found incidentally. Splenolymphangiomas are much rarer, commonly occur in children, and tend to have multiple foci. Splenic lymphangiomas are rare in adults, and fewer than 20 adult patients with isolated splenic lymphangiomas have been reported. In this article, we report the case of a middle-aged female patient with isolated splenic lymphangioma who underwent laparoscopic anatomical hypophysectomy of the lower pole of the spleen. We also summarize the existing literature on splenic lymphangioma diagnosis and available treatment options.

**Case presentation:**

A 58-year-old middle-aged woman was found to have a mass approximately 60 mm in diameter at the lower pole of the spleen during a health checkup that was not accompanied by other symptoms or examination abnormalities. After completing a preoperative examination with no contraindications to surgery, the patient underwent laparoscopic anatomical splenectomy of the lower extremity of the spleen. The patient recovered well without complications and was discharged from the hospital on the 7th postoperative day. Histopathological and immunohistochemical results confirmed the diagnosis of splenic lymphangioma. Prompt surgical intervention is safe and necessary when splenic lymphangiomas are large or associated with a risk of bleeding.

**Conclusion:**

Splenic lymphangiomas are rare and require early surgical intervention in patients with large tumor diameters or those at risk of rupture and bleeding. After rigorous preoperative evaluation and preparation, laparoscopic anatomical partial splenectomy is safe and feasible for surgeons with experience in laparoscopic surgery.

## 1 Introduction

Lymphangioma is a benign tumor, most commonly seen in children, occurring in the head, neck, and axillae (1). It may present as a lymphangioma syndrome, with the main pathological features of abnormally connected, dilated, and hyperplastic cystic lymphadenopathy (2). However, isolated lymphangiomas originating in the spleen are rare, and there is no standardized treatment protocol (2). We report a case of anatomical lower pole splenectomy for isolated splenic lymphangioma (1, 2). At the same time, we have summarized the existing literature and reviewed the diagnosis of splenic lymphangiomas, available treatment options, and prognosis.

## 2 Case presentation

**Figure 1:**
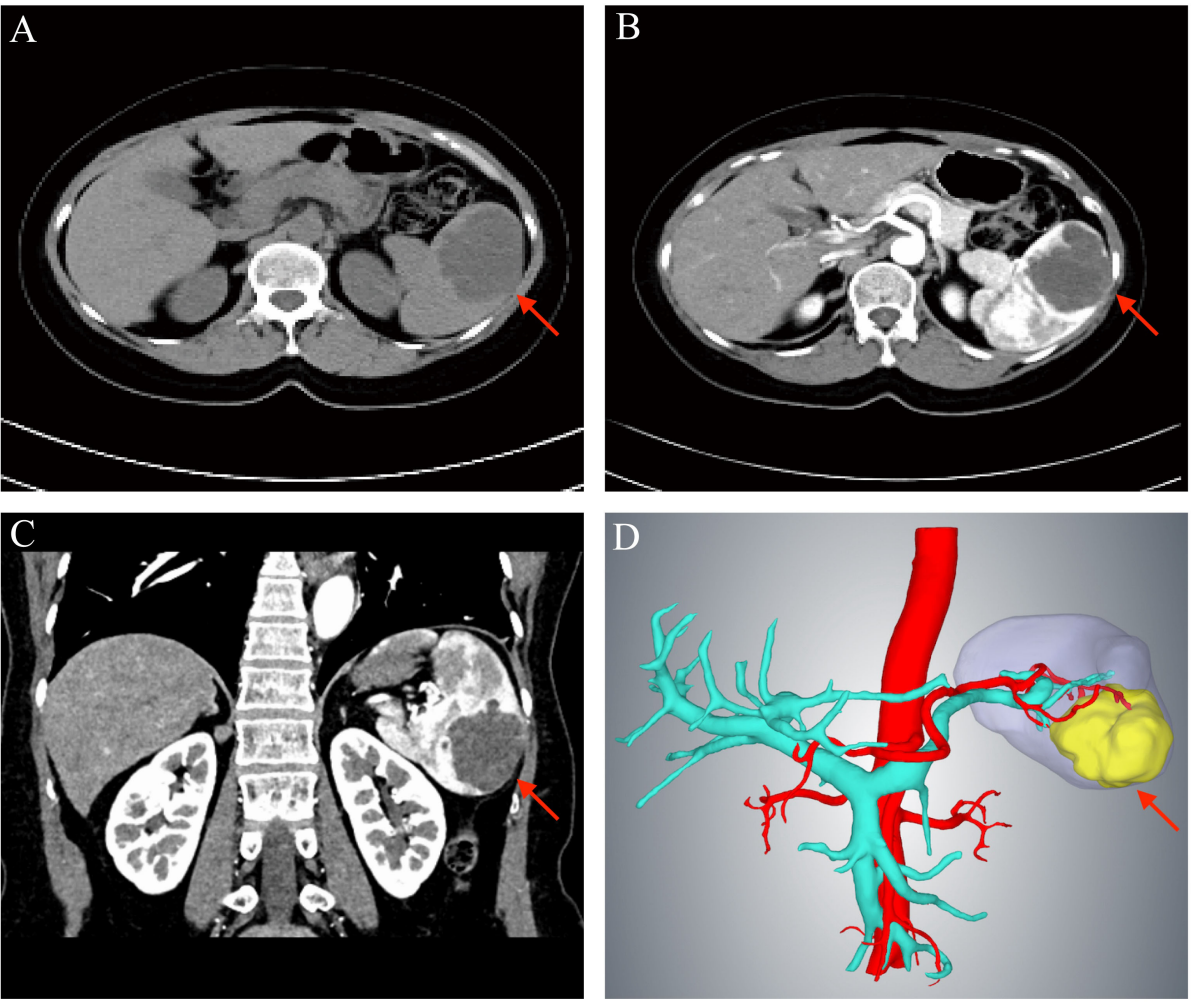
Preoperative examination of the patient. **(A)** Computed tomography scan showing a mass of about 61×15 mm in the lower pole of the spleen (red arrow). **(B)** Enhancement of the wall of the capsule with no obvious enhancement within the capsule is observed (red arrow). **(C)** The red arrow indicated the tumor. **(D)** Three-dimensional imaging showing the tumor confined to the lower pole of the spleen (red arrow) and supplied by the lower pole splenic artery.

A 58-year-old woman presented to our hospital with a splenic mass detected through abdominal ultrasonography performed during a health checkup. At the time of admission, the patient did not experience any discomfort such as abdominal pain, bloating, or nausea, and her body temperature, heart rate, respiratory rate, and body mass index were 36.4°C, 62 beats/min, 18 breaths/min, 104/59 mm Hg, and 23.03 kg/m^2^, respectively. Abdominal examination revealed no positive signs. The patient was previously fit and healthy, with no family history of hepatitis, cirrhosis, hematological disorders, or lymphatic disorders. Laboratory findings on admission were as follows: white blood cell count of 5.4 × 10^9^/L, red blood cell count of 4.59 × 10^12^/L, platelet count of 142 × 10^9^/L, and hemoglobin level of 131 g/L. Liver and renal function, electrolytes, serum tumor markers, coagulation function, and surface antigen levels of hepatitis B virus were all within normal range. Enhanced abdominal computed tomography (CT) revealed a mass measuring approximately 61 × 15 mm at the lower pole of the spleen, with enhancement of the wall of the capsule and no enhancement inside the capsule (**Figure 1**). Three-dimensional reconstruction suggested that the mass was confined to the lower pole of the spleen and supplied only by the lower pole splenic artery (**Figure 1**). Based on the patient’s medical history, laboratory test results, and abdominal CT findings, the mass was initially diagnosed as a benign splenic tumor. To further clarify the diagnosis and considering the large size of the mass and risk of rupture and hemorrhage, splenectomy was performed.

**Figure 2:**
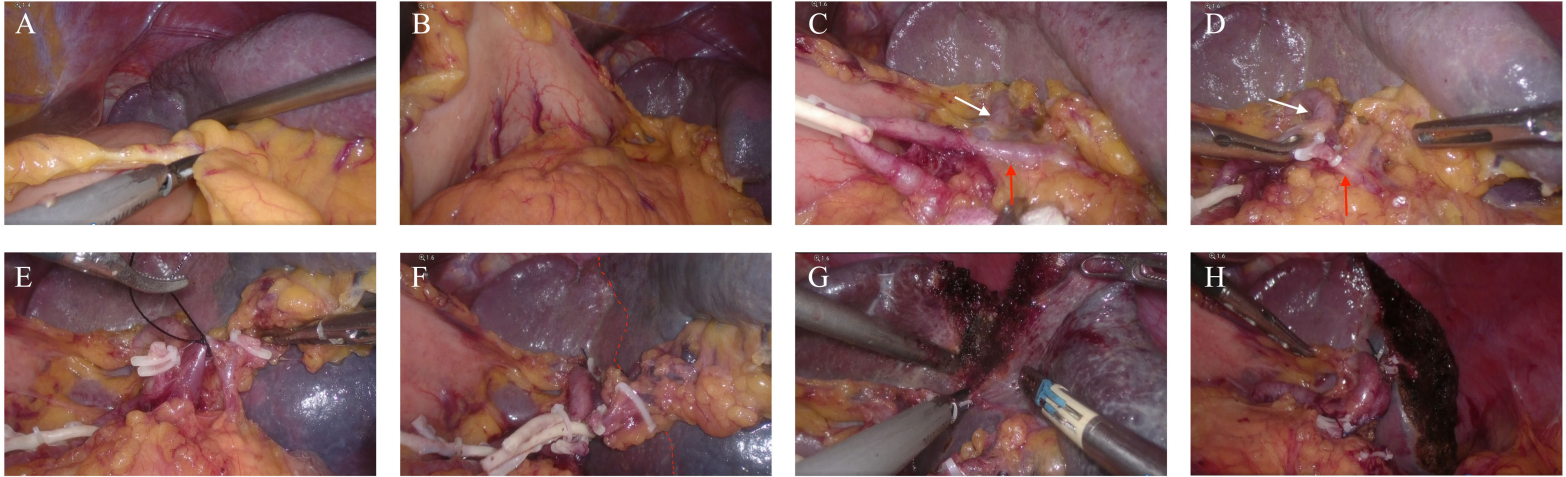
Intraoperative images. **(A, B)** Opening the gastrocolic ligament, suspending the stomach on the abdominal wall, and exposing the splenic hilum. **(C, D)** Naked inferior pole splenic artery and clamped for dissection, the red arrow indicated the inferior polar splenic artery and the white arrow indicated the superior polar splenic artery. **(E)** The inferior pole splenic vein branch is ligated. **(F)** Obvious ischemic line appearing after dissecting the inferior pole splenic vessel (red dotted line). **(G)** The spleen is dissected using an ultrasonic scalpel along the ischemic line 1 cm lateral to the spleen. **(H)** Cross-section of the spleen.

**Figure 3:**
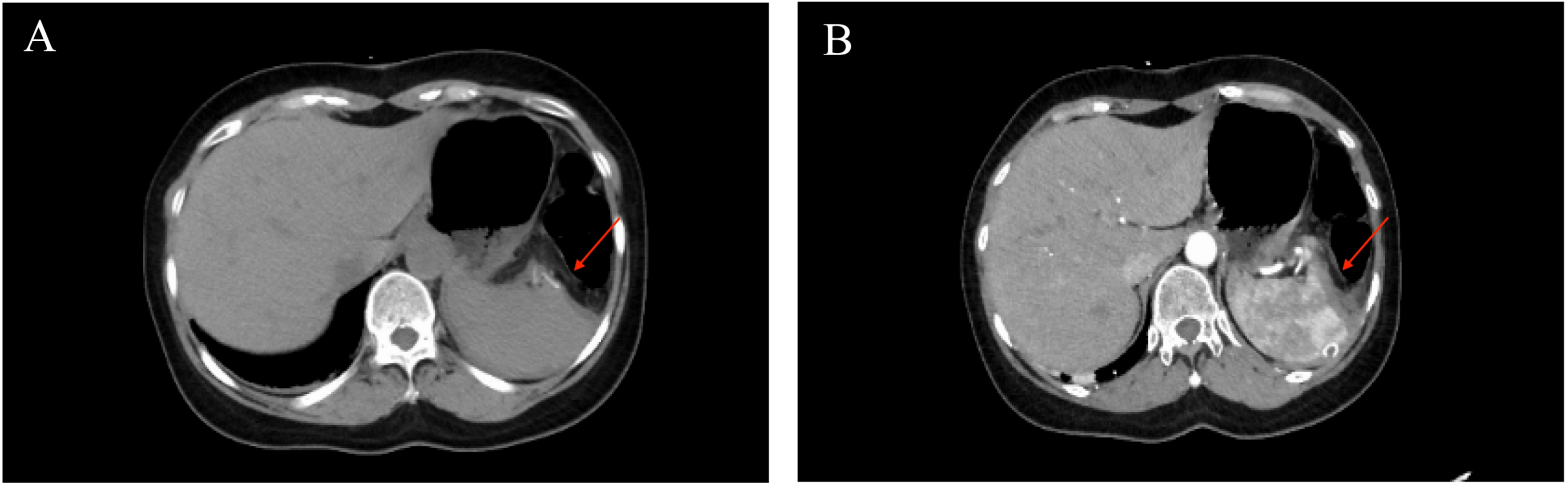
Postoperative computed tomography image. The red arrow **(A, B)** shows the spleen section, and no perisplenic blood or fluid accumulation was observed.

On the 3rd day after admission, laparoscopic anatomical lower-pole resection of the spleen was performed under general anesthesia. Intraoperative exploration revealed only an enlarged lower pole of the spleen, and no clear abnormalities were observed in other organs in the abdominal cavity. During surgery, we opened the gastrocolic ligament, suspended the stomach on the abdominal wall, exposed the splenic hilum, and naked the main trunk of the splenic artery. Subsequently, the inferior polar splenic artery and vein were excised and ligated. When the inferior polar splenic artery was ligated, a distinct ischemic line appeared on the splenic surface. Finally, splenic tissue was isolated along the ischemic the line on the surface of the spleen. After completely dissecting the lower pole of the spleen, the section was repeatedly rinsed and covered with an absorbable hemostatic fiber to ensure there was no obvious blood seepage (**Figure 2**). The entire operation lasted 140 min, with intraoperative bleeding of approximately 20 ml and a residual spleen volume of approximately 40——60%. The patient’s postoperative recovery was uneventful. Drainage from the splenic fossa was less than 10 ml/day for five consecutive days, and the drainage fluid was light red ascites. No perisplenic blood or fluid accumulation was observed on enhanced abdominal CT (**Figure 3**) on the 5th postoperative day, and the patient was discharged from the hospital on the 7th day after no abnormalities were found in the blood analysis, liver function, or renal function. The patient was followed up in the outpatient clinic for six months without any significant discomfort.

**Figure 4:**
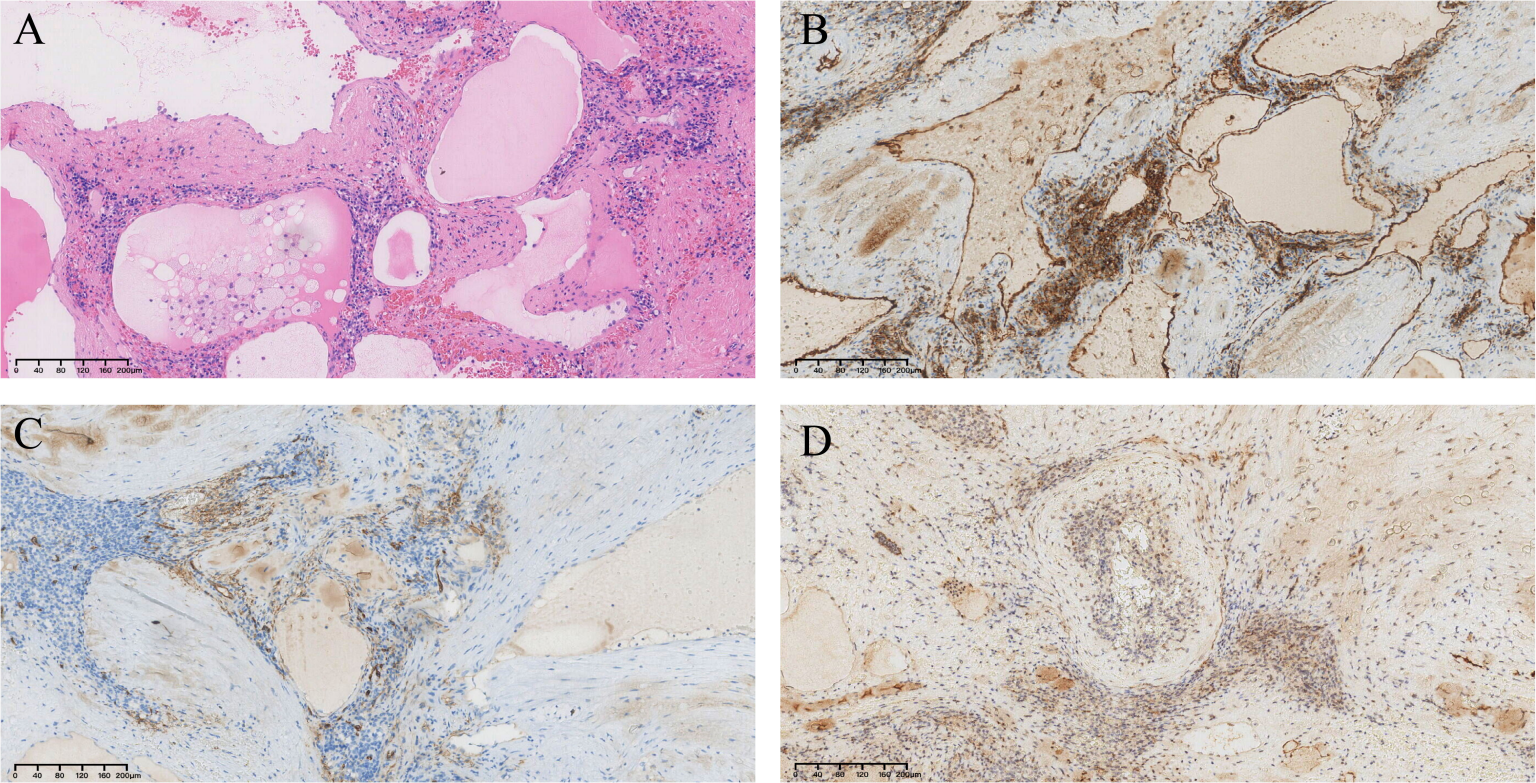
Pathological findings. **(A)**Hematoxylin and eosin staining shows a large number of cystic cavities in the middle of the spleen, filled with pink fluid and erythrocytes (×100). **(B)** CD31(+) (×100). **(C)** CD34 (partial +) (×100). **(D)** D2-40 (partial +) (×100).

Pathological findings revealed a large number of cystic cavities of different sizes in the middle of the tumor tissue, which were filled with powder-stained fluid and erythrocytes. The immunohistochemical staining results were as follows: D2-40 (partially +), CD31 (+), CD34 (partially +), P-CK (-), EMA (-), calretinin (-), WT-1 (-), and Ki-67 (+, approximately 2%) (**Figure 4**). The combined histopathological examination and immunohistochemical results led to a final diagnosis of splenic lymphangioma.

## 3 Discussion

Lymphangiomas tend to have a diffuse and multifocal distribution, often involving the liver, mediastinum, and lungs, resulting in lymphangioma syndrome (3). Splenic lymphangiomas were first reported by Frink et al. in 1885 (4). Isolated splenic lymphangiomas in adults are extremely rare and most often found incidentally. **Table 1** summarizes previous reports of isolated splenic hemangiomas in adults, along with treatment measures and outcomes (5–13).

**Table 1:** Previous reports on isolated splenic hemangiomas, treatment measures, and outcomes in adults.

Ref.	Age, yr	Sex	Diagnostic approaches	Size, mm	Symptoms	Operation	Outcomes
3	46	F	CT+MR	40	Dullness in the left upper quadrant of the abdomen	OTS	Recovered uneventfully during the one-year period follow-up
4	26	F	CT+MR	30	No relevant comorbidities	LTS	Follow-up six-month period, no complications were observed.
	30	F	CT	45	No relevant comorbidities	LTS	Follow-up six-month period, No complications were observed.
	40	F	CT	60	Accidental discovery of chronic cholecystitis	LTS+LC	Recovered uneventfully during the one-year period follow-up
5	67	M	CT+MR+PET	60	Accidental discovery of colon cancers	Not done	NM
6	30	F	CT	NM	Epigastric pain	OTS	NM
7	22	M	NM	42	NM	LPS	NM
	56	F	NM	40	NM	LPS	NM
	47	F	NM	44	NM	LPS	NM
8	51	F	CT+MR	15	Left upper abdominal discomfort	LPS	The patient was discharged on day 7 postoperatively with no complications.
9	64	F	CT+US	118	No relevant comorbidities	OTS	The patient was discharged 5 days later without complications
10	18	F	CT	60	Mild epigastralgia	LPS	The patient’s symptoms Disappeared with no recurrence at 1 year postoperatively
11	22	M	CT	NM	Post-traumaitc rupture bleeding	OTS	The patient after a follow-up of one year is asymptomatic
12	56	F	CT+MR	50	Chronic back pain	LTS	Perioperative course was uneventful and the chronic back pain resolved

F, female; M, male; CT, computed tomography; MR, magnetic resonance; PET, positron emission tomography; US, ultrasound; NM, not mentioned; LC, laparoscopic cholecystectomy; OTS, open total splenectomy; LTS, laparoscopic total splenectomy; LPS, laparoscopic partial splenectomy.

Patients with splenic lymphangiomas are typically asymptomatic and diagnosed upon physical examination. Currently, the diagnosis is mainly based on imaging studies, especially CT and magnetic resonance imaging (MRI). Typical CT characteristics of splenic lymphangiomas are single or multiple subperitoneal cysts with thin-walled margins and occasional enhancement of the peripheral wall. However, some solid cystic lymphangiomas may show significant enhancement of the parenchyma, but not of the capsule wall. On MRI, splenic lymphangiomas usually show a low signal on T1-weighted imaging but may show a high signal when the capsule is filled with proteinaceous material or accompanied by hemorrhage. On T2-weighted imaging, splenic lymphangiomas mainly show multicompartmental high signals corresponding to the dilated lymphatic vessels, with intervening fibrous connective tissues showing a low signal. Additionally, the high contrast of MRI may help detect rare malignant components within the capsule (14).

Pathological examinations and immunohistochemical findings remain the gold standard for confirming the diagnosis of splenic lymphangiomas. Splenic lymphangiomas characteristically appear microscopically as single or multiple cystic structures with macrophages, lymphocytes, and amorphous egg material in the cystic lumen. The surrounding tissues appear normal, congested, or fibrotic. Immunohistochemistry analysis for D2-40, CD31, CD34, factor VIII, and vascular endothelial growth factor receptor 3 may help confirm the diagnosis. D2-40 is a newly discovered monoclonal antibody that binds specifically to the endothelium of lymphatic vessels and is not expressed in the vascular endothelium, making it a highly sensitive and specific marker for the lymphatic endothelium (14). The immunohistochemistry results of our patient were positive for D2-40, along with CD31 and CD34; hence, the final diagnosis of splenic lymphangioma was confirmed.

Splenic lymphangiomas are rare, and there are no clear guidelines or consensus regarding splenic lymph nodes. Most scholars believe that the overgrowth of benign splenic tumors may lead to spontaneous rupture of the tumor or produce compression symptoms, so they should be surgically removed promptly. However, with advancing research on the physiological function of the spleen by scholars at home and abroad, more scholars have found that splenic insufficiency or hyposplenism may occur after total splenectomy, which may impair the patient’s immune system and anti-infective function. Total splenectomy will lead to the loss of patient resistance to bacilli, and although the chance of overwhelming posts-splenectomy infection (OPSI) can be reduced by preoperative prophylactic injection of *Streptococcus pneumoniae*, *Haemophilus influenzae*, and other immunological inoculants, there may still be some pathogens that are not covered by vaccines (15, 16). A previous study that included 1094 patients who underwent total splenectomy showed (17) that low autoimmune function after splenectomy lead to a significant increase in the risk of developing solid tumors, such as lung, ovarian, and liver cancers, as well as hematological malignancies.

Therefore, an increasing number of researchers have pointed out that partial splenectomy, under the premise of ensuring surgical safety, is conducive to maintaining immune function. A multicenter retrospective study pointed out that compared to patients who underwent total splenectomy, patients who underwent partial splenectomy did not suffer from postoperative complications such as serious infections, and their quality of life was not inferior to that of patients who underwent total splenectomy. Meanwhile, splenic volume regrew by an average of 15% (5——22%), suggesting that partial splenectomy can preserve the necessary splenic immune function and promote splenic regeneration while ensuring quality of life (9). In addition, Tripodiet et al. (18) demonstrated that there was no significant difference in the therapeutic efficacy of partial splenectomy compared to total splenectomy in the treatment of hereditary spherocytosis. This study also noted that although the incidence of short-term complications after partial splenectomy was higher than those after total splenectomy, this risk could be mitigated with the use of laparoscopic surgery, with statistically significant improvement only for postoperative blood transfusion (*P*=0.02). Therefore, partial splenectomy requires greater surgical proficiency, stricter awareness of minimally invasive procedures, and strict control of surgical indications. We have summarized the advantages and disadvantages of partial and total splenectomy in **Table 2**.

**Table 2:** Comparison of the advantages and disadvantages of partial and total splenectomy.

	PS	TS
Regenerative ability	Splenic volume regrew by an average of 15% (9).	Non-regenerative (9).
Postoperative quality of life	No significant difference (9).	
Incidence of OPSI	Preservation of partial anti-infective function reduces the incidence of postoperative OPSI (16).	TS can impair the patient's anti-infective function and induce OPSI (9, 17, 20).
Impact on immune function	Preservation of partial splenic immune function reduces the incidence of solid and hematologic malignancies (17).	Low autoimmune function after TS lead to a significant increase in the risk of developing solid and hematological malignancies (17).
Risk of recurrence of the original disease	Some studies have shown that there is no significant difference in postoperative recurrence of hereditary spherocytosis between PS and TS (18).	Postoperative recurrence of some hematologic diseases may be one of the primary reason for conversion to total splenectomy (22).
Surgical complexity	The intricate vascular architecture of the spleen poses significant challenges to intraoperative manipulation and hemostasis (20).	Ligation of the splenic pedicle alone is sufficient, resulting in relatively lower surgical complexity (20).
Surgical indications	Surgical indications must be carefully evaluated. The decision needs to be based on the patient's specific situation, the nature and extent of the splenic lesion, the patient's overall health, and the experience of the operator (21).	It is applicable to nearly all patients requiring splenectomy (21).

PS, partial splenectomy; TS, total splenectomy; OPSI, overwhelming post-splenectomy infection.

By directly ligating the blood-supplying vessels of the lower pole of the spleen, sufficient splenic parenchyma was preserved, while effectively controlling intraoperative bleeding, which was conducive to preserving the patient’s splenic function and postoperative splenic regeneration. Notably, owing to the complexity of the splenic blood supply, intraoperative bleeding management is essential to the outcome of this procedure. In recent years, several researchers have proposed different methods to control intraoperative bleeding, such as anatomical splenic vascular ligation, preoperative embolization or radiofrequency ablation (19), hand-assisted laparoscopic partial splenectomy (20), and robot-assisted partial splenectomy, which are all effective in reducing intraoperative bleeding. Currently, the most commonly used approach is still partial splenectomy through a revealed ischemic line after blocking the splenic blood supply, and applying bipolar electrocoagulation, ultrasound devices, unipolar thermotherapy, and hemostatic substances to the splenic section to reduce blood seepage (21). Following this concept, we used 3D reconstruction to understand the distribution of splenic vessels, clamped the blood-supplying vessels of the lower pole of the spleen, revealed the splenic ischemic line, dissected the spleen along the ischemic line, and used bipolar electrocoagulation to strictly stop bleeding while dynamically monitoring the surface of the incision to prevent uncontrolled blood seepage from the small branching vessels. After complete dissection of the lower pole of the spleen, the wound was repeatedly inspected and covered with a hemostatic fiber. The patient recovered well and was discharged from the hospital on the 7th postoperative day.

## 4 Conclusion

Laparoscopic anatomical partial splenectomy for benign splenic tumors, such as isolated lymphangiomas, is safe and feasible for surgeons experienced in laparoscopic surgery with adequate preoperative evaluation and preparation.

## Data Availability

The original contributions presented in the study are included in the article/supplementary material. Further inquiries can be directed to the corresponding authors.
